# Seasonality Drives Microbial Community Structure, Shaping both Eukaryotic and Prokaryotic Host–Viral Relationships in an Arctic Marine Ecosystem

**DOI:** 10.3390/v10120715

**Published:** 2018-12-14

**Authors:** Ruth-Anne Sandaa, Julia E. Storesund, Emily Olesin, Maria Lund Paulsen, Aud Larsen, Gunnar Bratbak, Jessica Louise Ray

**Affiliations:** 1 Department of Biosciences, University of Bergen, N-5020 Bergen, Norway; julia.storesund@hi.no (J.E.S.); eolesin@gmail.com (E.O.); maria.l.paulsen@uib.no (M.L.P.); aud.larsen@uib.no (A.L.); gunar.bratbak@uib.no (G.B.); jessicalouiseray@gmail.com (J.L.R.); 2 NORCE Norwegian Research Centre AS, Uni Research Environment, N-5020 Bergen, Norway

**Keywords:** arctic, marine, myovirus, algal viruses, metabarcoding, MCP, g23, polar winter, succession

## Abstract

The Arctic marine environment experiences dramatic seasonal changes in light and nutrient availability. To investigate the influence of seasonality on Arctic marine virus communities, five research cruises to the west and north of Svalbard were conducted across one calendar year, collecting water from the surface to 1000 m in depth. We employed metabarcoding analysis of major capsid protein *g23* and *mcp* genes in order to investigate T4-like myoviruses and large dsDNA viruses infecting prokaryotic and eukaryotic picophytoplankton, respectively. Microbial abundances were assessed using flow cytometry. Metabarcoding results demonstrated that seasonality was the key mediator shaping virus communities, whereas depth exerted a diversifying effect within seasonal virus assemblages. Viral diversity and virus-to-prokaryote ratios (VPRs) dropped sharply at the commencement of the spring bloom but increased across the season, ultimately achieving the highest levels during the winter season. These findings suggest that viral lysis may be an important process during the polar winter, when productivity is low. Furthermore, winter viral communities consisted of Operational Taxonomic Units (OTUs) distinct from those present during the spring-summer season. Our data provided a first insight into the diversity of viruses in a hitherto undescribed marine habitat characterized by extremes in light and productivity.

## 1. Introduction

Global climate change is accelerating at higher latitudes, potentially threatening vulnerable Arctic marine ecosystems [1]. Our current understanding of Arctic ecosystems is one regulated by bottom-up control and impacted by high seasonal contrasts in light and primary productivity. Such contrasts imply a microbial community adapted to extremes in light and resource availability, ranging from constant light and high productivity during the spring-summer period to constant dark and low productivity during the polar winter. However, this understanding is mostly based on biological surveys conducted during the light period. In order to achieve a more precise understanding of Arctic ecosystems, we must also include investigation of the underexplored polar night, particularly as unexpected levels of biological activity have been observed among zooplankton during the winter [2,3]. Whether this also applies to lower trophic levels is still an open question, but as Arctic regions are experiencing accelerated environmental change, the question is urgent and calls for an answer.

There is now a considerable body of evidence suggesting that microbial diversity and abundance vary in step with environmental changes in high latitude marine ecosystems [4,5,6]. Less, however, is known about Arctic marine viral communities, where host specificity plays a vital role in regulating microbial diversity and nutrient recycling. Being the most numerous and diverse biological entities in the oceans [7,8], viruses are one of the main drivers of the marine microbial food web (MMFW), affecting both host diversity [9,10,11] and the flow of nutrients [8,12] in the ocean. Viral communities in Arctic and temperate waters are large, active, and diverse [13,14,15], with as much as 20–40% of primary production flowing through the viral shunt of the microbial loop. Through their lytic activity, viruses affect the function of the microbial food web by altering host communities both through increased genetic drift [16] and altered community structure as a consequence of the arms race between viruses and their hosts [17]. Further, viruses are also involved in the transformation of organic matter between microbial biomasses and the dissolved organic carbon pool [18,19]. The release of organic matter through the viral shunt has been estimated to be 150 billion tons of organic carbon per year [20]. Recently, a strong correlation between viral activity and carbon export by sinking particulate matter was observed in an oligotrophic ocean [21]. This indicated that viruses significantly contribute not only to the structuring of host communities, nutrient release, and ecosystem productivity, but also to the downward vertical transport of particulate carbon that comprises the biological carbon pump [22].

For viruses, unlike cellular organisms, there is no universal marker gene that targets all viruses [23]. Viral group-specific genes, however, can capture elements of diversity for ecologically important virus groups. In the marine environment, common viral groups that are targeted for molecular analysis include bacteriophages (*g20* and *g23* genes), cyanophages (*psdA* and *psdD* genes), and the large double-stranded DNA phytoplankton viruses (Major Capsid Protein (MCP) and DNA polymerase genes) (summarized in Reference [24]). Phylogenetic analysis of these viral genes has evidenced an enormous genetic diversity among marine viruses. Most recently, metabarcoding analysis of key viral groups such as T4-like myoviruses and algal viruses has greatly increased insight into the structure and function of marine viral assemblages [25,26]. T4-like phages belongs to the Myoviridae family, a group of lytic phages with genetic homology to the coliphage T4 [27]. This phage group is one of the most diverse, widespread, and abundant phage groups in marine environments [28,29,30]. Cultured T4-like Myoviridae infect a wide range of phylogenetically distant heterotrophic bacteria such as *Vibrio*, *Pseudomonas*, and SAR11 [31]. They also infect autotropic prokaryotes, such as *Prochlorococcus* and *Synechococcus*, with the main fraction of hereto-isolated cyanophages belonging to this group [31]. Most described isolated viruses infecting picoeukaryotes (heterokonts, haptophytes, and cryptophytes) belong to the families Phycodnaviridae [32] and Mimiviridae [33]. They are also widely distributed and abundant in the global ocean [26,34,35]. Many hosts of these viruses are key species in the Arctic environment (e.g., the spring bloomer *P. pouchetii* [36] and the prasinophyte *Micromonas*), the most abundant taxon reported in the Arctic from summer to autumn [37,38]. MCP primers capturing both algal viruses within the Megaviridae family [26] (including viruses that infect haptophytes such as *Phaeocystis pouchetii*, *Phaeocystis globosa*, *Prymnesium kappa*, and *Haptolina ericina*) and *Prasinoviruses* within the Phycodnaviridae family have been successfully applied to studies of virus ecology in high-latitude marine environments [26].

In order to expand our insight into viral ecology in Arctic marine environments, we conducted a metabarcoding study of seawater viral diversity and abundance from virus assemblages collected across one calendar year and at depths ranging from the surface to 1000 m. Our metabarcoding approach targeted genes for the two key viral groups: The *g23* gene of T4-like myoviruses [28,29] and the *mcp* gene of large algal dsDNA viruses [26]. By targeting these key and diverse viral groups, regulating both heterotrophic and autotrophic prokaryotes and eukaryotic phytoplankton, we investigated one major top-down regulatory element of dominant microbial assemblages in periods of high productivity, but also in periods of low productively (i.e., the underexplored polar winter and the deep ocean). As light and depth have previously been shown to be key for the regulation of other microbial constituents (prokaryotes and protists) [4,38], a natural extension from these findings is that co-occurring viral assemblages also experience seasonal dynamics. Results from our molecular diversity analysis were compared to flow cytometry counts of viruses, prokaryotes, and eukaryotic pico- and nanophytoplankton.

## 2. Materials and Methods

### 2.1. Sampling and Sample Preparation

**Figure 1:**
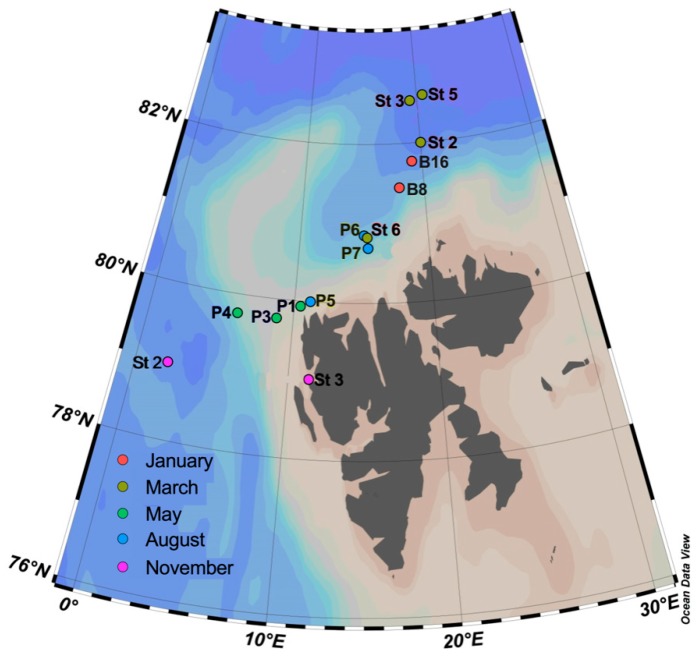
Sampling stations during the five cruises performed in 2014. Symbol colors are uniform for sampling month, whereas station labels indicate the names of sampling stations. The same color scheme is utilized throughout this study.

Water samples were collected during five cruises west and north of the Svalbard archipelago in 2014, as described in detail in Paulsen et al. [39] (Figure 1). Transects across the core of Atlantic water (AW) inflow were made at 79° N and 79.4° N during May (15.05–02.06), August (07.08–18.08), and November (03.11–10.11). On the January (06.01–15.01), March (05.03–10.03), and August cruises, samples were collected from the southern branch of the Western Svalbard Current (WSC), which transports water into the Arctic Ocean (80.5 to 82.6° N). The choice of sampling area and stations was largely determined by the extent of the sea ice (Paulsen et al., 2016) [39]. Vertical profiles of temperature, salinity, and fluorescence were recorded on each sampling occasion using an SBE 911plus CTD system (Sea-Bird Scientific USA, Bellevue, WA, USA). A detailed overview of physical and chemical parameters for the water samples investigated can be found in the ENA (https://www.ebi.ac.uk/ena/data/view/PRJEB17856) and the PANGAEA Data Publisher for Earth and Environmental Science (https://doi.pangaea.de/10.1594/PANGAEA.884255).

In total, 42 samples were collected from 3–4 different depths per station, ranging from 1 to 1000 m (Table S1) at each sampling station, using 10 L Niskin bottles. Different water masses were targeted according to the definitions provided in Paulsen et al. [39]. The shallow shelf stations were sampled to near-bottom, whereas deeper stations were sampled down to 1000 m. Samples were given names indicating “month of sampling_sampling station_sampling depth in meters (m)” (e.g., “Jan_B8_1000” indicates the sample taken in January at station B8 and at a 1000 m water column depth). Samples for flow cytometric enumeration of prokaryotes and viruses were preserved with 1% (*v*/*v*) glutaraldehyde for 30 min at 4 °C in the dark [40]. Samples were then snap-frozen and stored in liquid nitrogen until counting on board the research vessel (see below).

For molecular analysis, 50 L of seawater were pre-filtered through 3 µm and 0.45 µm low-binding filters (Durapore, Millipore, Burlington, MA, USA), with the exception of 5 samples (Mars_S2_1000, Mars_S6_1000, May_P4_1000, Aug_P6_1000, Aug_P7_1000), for which 0.2 µm filters were used instead of 0.45 µm. Filtration was performed using a tandem setup with stainless steel standing 142 mm filter holders (Merck-Millipore) and gentle peristaltic pumping to remove larger particles. The filtrate containing virus particles was concentrated to 50–100 mL using a tangential flow filtration (TFF) system equipped with a 100,000 pore size (NMWC) hollow-fiber cartridge (QuixStand, GE Healthcare Bio-Sciences AB, Uppsala, Sweden). Aliquots of the resulting virus concentrates were snap-frozen in liquid nitrogen on board the research vessel, then transferred on dry ice to −80 °C storage in the laboratory.

#### Flow Cytometry (FCM)

FCM analysis were performed using an Attune ^®^Focusing Flow Cytometer (Applied Biosystems by Life Technologies) with a syringe-based fluidic system and a 20 mW 488 nm (blue) laser. The samples were fixed with glutaraldehyde (0.5% final conc.) at 4 °C for a minimum of 30 min, flash frozen in liquid nitrogen and stored at −80 °C until analysis, except in November where autotrophic pico- and nanoplankton were enumerated in fresh samples. For autotrophic pico- and nanoplankton the trigger was set to chlorophyll *a* autofluorescence enabling us to identify three different populations of autotrophic phytoplankton according to variation in side scatter, chlorophyll *a* and phycoerythrin autofluorescence: the cyanobacterium *Synechococcus*, picophytoplankton and nanophytoplankton. Samples for enumeration of virus-like particles (VLP), prokaryotes and heterotorphic nanoflagellates (HNF) were thawed rapidly, and for VLP and prokaryotes appropriately diluted, before staining with SYBR Green I (Molecular Probes, Eugene, OR, USA, 10^−4^ of commercial stock). Samples for enumeration of VLP and prokaryotes were stained for 10 min in an 80 °C water bath according to [40] whereas HNF samples were enumerated after a 2 h staining in the dark following recommendations of [41]. Samples were counted with green fluorescence as trigger, using a flow rate of 25 μL min^−1^ for prokaryotes and VLP, and 500 μL min^−1^ for HNF. Gates for prokaryotes and three different virus populations were defined based on side scatter properties and green fluorescence: low, medium and high fluorescence viruses (small, medium and large) respectively [42,43]. A minimum of 1mL was analyzed for HNF enumeration, and the population was discriminated from nano-sized phytoplankton based on green vs. red fluorescence and from large bacteria using a plot of side scatter vs. green fluorescence [44].

Virus-to-prokaryote ratios (VPRs) were calculated by dividing small virus abundance by heterotrophic prokaryote abundance. The significance of sampling month and water column depth on the variation in virus and prokaryote abundances and virus-to-prokaryote ratios was assessed using generalized linear models (glm in the base package stats) in RStudio v.1.1.423 (Boston, MA, USA). We used a Gaussian distribution and identity link function in which log_10_-transformed counts/VPR were response variables, whereas sampling month and depth were explanatory variables. The parameter test = “Chisq” was added to provide *p*-values.

### 2.2. DNA Extraction and High-Throughput Sequencing Library Construction

Viral particles in 1–2 mL virus concentrates were first disrupted using two cycles of heating to 90 °C for 2 min, then chilled on ice for 2 min. Disodium ethylenediamenetetraacetic acid (EDTA) and Proteinase K were added to a final concentration of 20 mM and 100 µg mL^−1^, respectively, before the samples were incubated for 10 min at 55 °C. Sodium dodecyl sulphate (SDS) was then added to a final concentration of 0.5% (*w*/*v*), and the samples were incubated for an additional 1 h at 55 °C. Double-stranded DNA was then purified from lysates using the Zymo DNA Clean and Concentrator−5^TM^ kit (Zymo Research, Irvine, CA, USA) according to the manufacturer’s protocols.

In total, 40 g23 and 32 MCP libraries were sequenced using Illumina MiSeq paired-end sequencing, while 8 additional MCP libraries (January samples) were sequenced by unidirectional Roche/454 sequencing. Partial fragments of the *g23* and *mcp* genes were amplified from DNA preparations as described previously [26,28,29]. Eight replicate PCR reactions were run per sample. The replicates were pooled and purified using Agencourt AMPure XP Beads (Beckman Coulter Inc., Brea, CA, USA). Secondary amplifications of the pooled amplicons were primed by MID-tagged primers (Illumina, San Diego, CA, USA) or a MID-tagged forward primer in combination with a Lib-L-adapter A reverse primer (Roche/454, Basel, Switzerland). PCR products from second amplifications were purified (Agencourt AMPure XP Beads, Beckman Coulter Inc., Brea, CA, USA), quantified, and pooled in equimolar amounts to generate Illumina or 454 libraries. Libraries were sequenced on an Illumina MiSeq platform using v3. PE300 sequencing chemistry or the Roche/454 pyrosequencing GS Titanium platform and Gold reaction (GS20, Roch/454 Life Sciences) at the Norwegian Sequencing Centre in Oslo, Norway (https://www.sequencing.uio.no/) (NSC, January samples) or the University of California Davis DNA Sequencing Facility (http://dnaseq.ucdavis.edu/SampleSubmission.cfm) (UCD, March, May, August, and November samples). Demultiplexed sequence reads were stored at the NIRD Research Data archive and are publicly available as a Sequence Read Archive through the European Bioinformatics Institute (accession number PRJEB17856).

### 2.3. Sequence Analysis

Adapters and phiX contamination were removed from Illumina paired-end reads, merged, and quality-trimmed with a minimum cutoff of 20, using bbduk and bbmerge (BBMap-35.07 suite, https://sourceforge.net/projects/bbmap). Fastq files were converted to fasta files using QIIME v.1.9.1 [45], and 454 sequence reads were quality-checked and trimmed using a combination of mothur v.1.36.1 [46] and QIIME. De novo chimera detection and OTU clustering at 97% sequence similarity was performed on the g23 Illumina reads, and on the combined Illumina and 454 MCP reads using USEARCH [47] as implemented in mothur. Affiliations of reads were assigned in mothur using custom-made databases of 1597 g23 and 25 MCP sequences as reference, obtained from Genbank (June 2016). In total, 3,142,382 sequence reads clustering into 5002 OTUs were generated for the *g23* gene, while 1,094,759 sequence reads clustering into 189 OTUs were generated for the *mcp* gene. To see if sequence method affected the observed OTU diversity, an ANOVA test was done on the beta diversity of the samples (function betadisper, vegan package [48]). We did not observe any significant differences in OTU diversity generated by 454 versus MiSeq sequencing (*p* = 0.38) (Figure S1), and therefore sequence results from the two platforms were combined for all statistical analyses.

All statistical analyses were performed in the R statistical computing environment (R Core Team, 2017) using the vegan v2.4.5 [48], ggplot2 v3.0.0 [49], and gplots v3.0.1 ([50]). For g23, the OTU table was subsampled to the sample with the lowest number of reads per sample (6079 reads) for all analyses except rarefaction. Relative abundances of each g23 OTU in the subsampled dataset were used for further analysis. For MCP, the lower number of reads generated by 454 relative to Illumina sequencing would have resulted in subsampling to 304 sequences per sample, significantly reducing the size of the dataset utilized for statistical analysis. To avoid this data reduction, all statistical analyses of MCP were therefore based on the relative abundances of each OTU in the individual samples without subsampling.

Differences in Shannon diversity (H’) as a function of depth, sampling time (month), station, latitude, salinity, and water mass were tested using ANOVA on linear models. In order to determine if g23 or MCP community composition differed between sampling stations, depth, water mass, or sampling month, multivariate dispersions were calculated and subjected to ANOVA tests. From these tests, nonmetric multidimensional scaling (NMDS) and canonical correspondence analysis (CCA) plots were constructed to illustrate sample distribution. Significant correlations of changes in community structure (CCA) with environmental variables were determined using ordistep (vegan). VPR was log_10_-transformed prior to CCA analysis. For a few samples where chlorophyll *a* values were lacking, we interpolated values from the surrounding samples in order to avoid NA errors for CCA analysis. A constant was added to all sample results having zero values in order to allow log transformation. To visualize the dynamics of dominant OTUs (i.e., OTUs comprising >0.1% of the entire dataset) over time and at different depths, heat maps were constructed. In total, 84 OTUs and 29 OTUs were used to construct heat maps for g23 and MCP, respectively. Briefly, distance matrices (Euclidian distances) were constructed in R using the function vegdist (vegan). This was followed by hierarchical clustering analyses of the dissimilarity matrixes using average linkage clustering. The resulting cluster dendrograms were used to construct heat maps using the heatmap.2 function in the gplots package.

To construct a phylogenetic tree containing the dominant MCP OTUs, nucleotide sequences were first translated to amino acid sequences using BLAT v.35 [51] and aligned together with 25 reference sequences representing isolated and characterized viruses using MUSCLE v.3.8.31 [52]. Maximum likelihood trees (100 bootstraps) were constructed in MEGA v.6 [53]. For g23, the underrepresentation of cultivated myovirus isolated sequences in the reference databases, in combination with the high genetic diversity of this gene, precluded our ability to extract meaningful results about keystone species from phylogenetic analysis of this marker gene.

## 3. Results

### 3.1. Microbial Abundance

#### 3.1.1. Single-Celled Eukaryotes and Cyanobacteria

**Figure 2:**
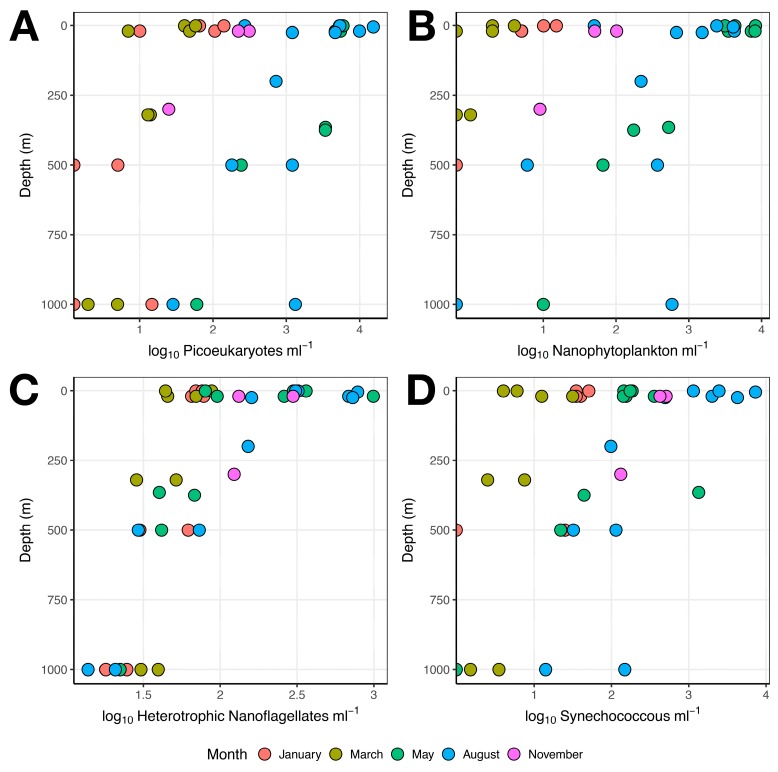
Scatterplot showing abundances of key eukaryotic microbial populations as determined by flow cytometric enumeration. (**A**) Pico, (**B**) nano, (**C**), HNF, and (**D**) cyanobacteria. Depth (m) is shown on the *y* axis. Sampling months are represented with different colors. Note logarithmic *x* axis.

FCM counts of microbial autotrophs and heterotrophic nanoflagellates (HNF) revealed strong season- and depth-driven differences in population density. For all four groups examined (picophytoplankton, nanophytoplankton, HNF, and cyanobacteria), we observed a three- to four-order of magnitude difference in population densities across the sampling year and along the depth profile, with the highest densities observed in surface waters in May and August (~10^4^ cells mL^−1^), and the lowest densities observed in January and March and at depth (0–~10^2^ cells mL^−1^) (Figure 2). November samples indicated a transitional phase between summer and winter microbial communities, with intermediate surface abundances for all microbial eukaryote groups assessed (Figure 2). May and August samples exhibited high population densities even at depths down to 500 m (Figure 2). Samples taken during January and March had stable low populations of microbial eukaryote population densities at all depths (Figure 2). Generalized linear models (model = glm, family = Gaussian) of log_10_-transformed flow cytometry counts as response variables and depth + month as explanatory variables identified significant (Pr(>|t|) < 0.05) effects of both depth and month on the variation in abundance of picoeukaryote (null deviance = 58.64 on 39 DF, residual deviance = 8.80 on 34 DF, AIC 66.95), nanoeukaryote (null deviance = 233.31 on 39 DF, residual deviance = 42.69 on 34 DF, AIC 130.12) and *Synechococcus* sp. (null deviance = 43.80 on 39 DF, residual deviance = 8.61 on 34 DF, AIC 66.08) assemblages.

#### 3.1.2. Heterotrophic Prokaryotes (HPs)

**Figure 3:**
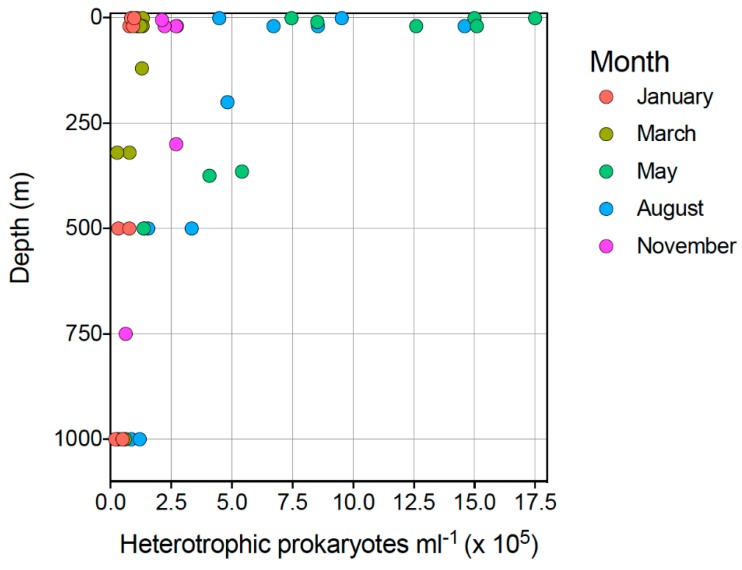
Scatterplot showing abundances of heterotrophic prokaryotes (HPs) as determined by flow cytometric enumeration. Depth (m) is shown on the *y* axis. Sampling months are represented with different colors. Note logarithmic *x* axis.

The highest counts of heterotrophic prokaryotes were observed in surface waters (≤25 m) in May and August (1.26–1.75 × 10^6^ HP mL^−1^), while the lowest abundances were observed at depth (>300 m) in March (2.75–7.79 × 10^4^ cells mL^−1^, *N* = 4) and January (1.97–7.72 × 10^4^ cells mL^−1^, *N* = 4). Low abundances were also observed in May (4.87 × 10^4^ HP mL^−1^) and August (8.53 × 10^4^ HP mL^−1^) (both >300 m) (Figure 3). This revealed a variation in HP abundance of approximately 100-fold across one sampling year and 1000 m in water column depth. A generalized linear model with log_10_-transformed HP mL^−1^ as a response variable and depth + month as explanatory variables identified a significant effect of both depth and month on HP counts (model = glm, family = Gaussian, null deviance = 12.53 on 39 DF, residual deviance = 1.84 on 34 DF, AIC 4.34). The model further identified two groupings of HP abundances based on sampling month: May-August and January-March-November. HP abundances were in general higher in May-August than in January-March-November regardless of sampling depth. Prokaryote abundance was weakly yet significantly negatively correlated with sampling depth (Pearson’s *r* = −0.49, t = −3.46, *p*-value = 0.0014).

#### 3.1.3. Viruses

**Figure 4:**
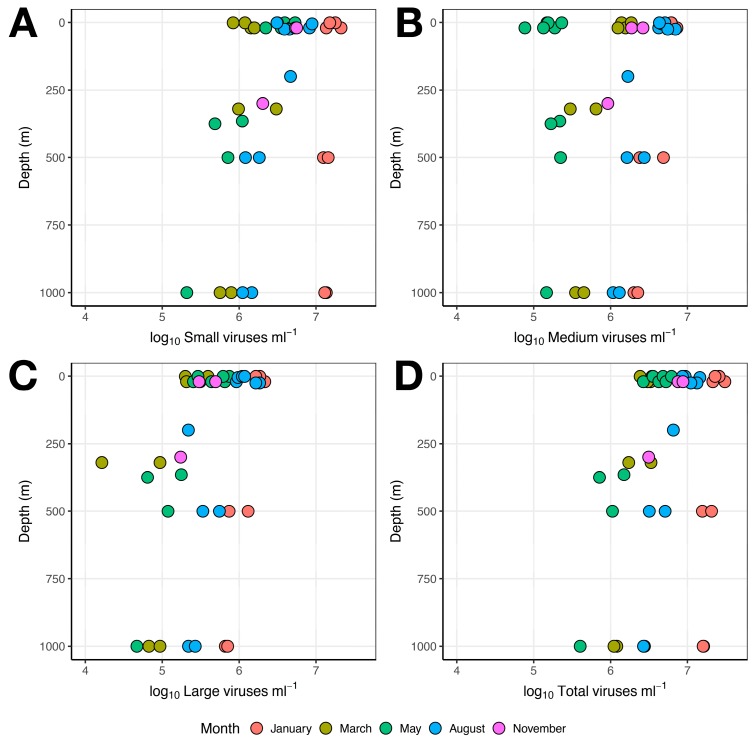
Scatterplot showing abundances of virus-like particles (VLPs) as determined by flow cytometric enumeration. (**A**) Small viruses, (**B**) medium viruses, (**C**) large viruses, (**D**) total viral abundance. Depth (m) is shown on the *y* axis. Sampling months are represented with different colors. Note logarithmic *x* axis.

Flow cytometry revealed that small viruses accounted for the majority of VLPs present in the samples analyzed (Figure 4). Grouped by sampling month, small virus flow cytometry counts were highest in August (3.05 ± 2.02 × 10^6^ virus-like particles mL^−1^) at all sampling depths. The lowest abundances of small viruses were observed in deeper water layers (>300 m) in May and August (1.02 ± 0.53 × 10^6^ VLP mL^−1^) and in all water layers in March (1.30 ± 0.78 × 10^6^ VLP mL^−1^) (Figure 3). Small virus VLP abundances thus ranged from 2.09 × 10^5^ VLP mL^−1^ (May, 1000 m depth) to 8.24 × 10^6^ VLP mL^−1^ (August, 20 m depth), a difference from lowest to highest abundance of approximately 40-fold. Both sampling month (*Pr*(>*Chi*) < 0.001) and depth (*Pr*(>*Chi*) = 0.0012) accounted for the observed variation in small VLP abundances (model = glm, family = Gaussian, null deviance = 4.8214 on 39 DF, residual deviance = 1.9191, AIC = 6.0348). The highest abundances for medium (3.23 ± 1.65 × 10^6^ VLP mL^−1^) and large (7.55 ± 4.86 × 10^5^ VLP mL^−1^) viruses were detected in the August samples (Figure 4). In general, medium and large viruses followed the same depth-driven trend as small viruses, with the highest VLP abundances in surface water and decreasing VLP abundance with increasing depth. Medium VLPs in May did not follow this trend, as similar abundances were detected at all depths (Figure 4). Total VLP abundance was weakly yet significantly negatively correlated with sampling depth (Pearson’s *r* = −0.504, t = −3.60, *p*-value = 0.0009112).

**Figure 5:**
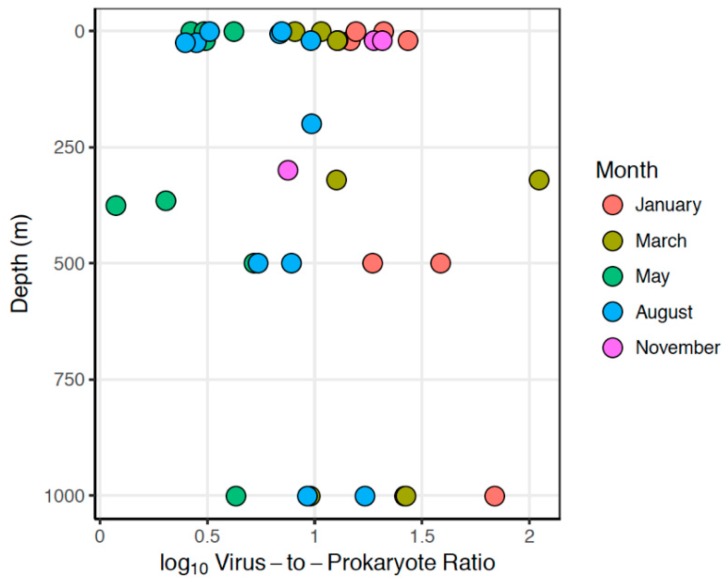
Virus-to-prokaryote ratios (VPRs). VPRs were calculated by dividing flow cytometry counts of small viruses (V1) by flow cytometry counts of heterotrophic prokaryotes (HPs). Depth (m) is shown on the *y* axis. Sampling months are represented with different colors.

Small viruses in the ocean likely represent bacteriophages [43] whose host populations consist primarily of heterotrophic bacteria (Reference [42] and references therein). In order to investigate relative changes in host and virus abundances and thus assess host–virus dynamics, we compared flow cytometric counts of heterotrophic prokaryotes (HPs) and small VLPs as one potential host–virus assemblage in the Arctic marine pelagic environment (Figure S2). When virus-to-prokaryote ratios (VPRs) were calculated from flow cytometry counts of small VLPs and HPs, we observed the highest VPRs in the March samples (VPR = 8.1–110.5), while the lowest VPRs were observed in the May samples (1.2–5.2) (Figure 5). Similarly to both small VLP and HP abundances, VPR was significantly influenced by both sampling month and sampling depth, with the strongest effect from sampling month (*p* < 0.001). More specifically, the influence of sampling month on VPR calculations resulted in two groupings: January-March-November and May-August. VPRs were considerably lower in the May-August group relative to the January-March-November group.

### 3.2. Sequence Analysis

After quality trimming and chimera removal, 9.4% to 68.1% of the g23 and 12.8% to 42.5% of the MCP reads remained (Table S1). For the MCP reads it was not possible to calculate the percentage of reads after trimming and quality checking, as the data was not demultiplexed until after trimming and quality checking (Table S1). OTU clustering with 97% identity gave 7–36 unique OTUs for the MCP and between 69–566 unique OTUs for the g23 gene (Table S1).

**Figure 6:**
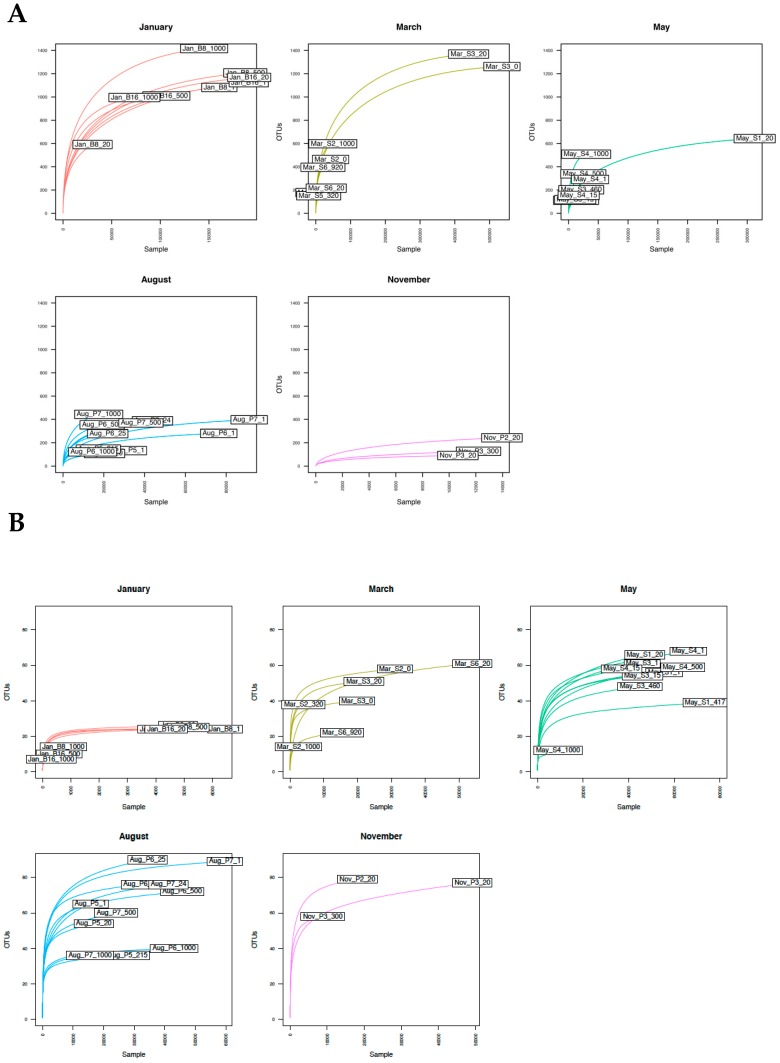
Rarefaction analysis of (**A**) g23 and (**B**) MCP OTU diversity. OTUs with a relative abundance >0.1% were included.

Rarefaction analysis of OTU richness for g23 (Figure 6A) and MCP (Figure 6B) virus assemblages demonstrated opposite trends in genetic richness across sampling months. T4-like myoviruses in the January samples at all depths showed high rarefied richness, indicating a rich genetic diversity with sequencing depth approaching saturation for all samples, with the exception of Jan_B8_20. Genetic diversity in the March samples remained high in only two surface samples taken at St. 3, Mar_S3_20 and Mar_S3_0. The genetic diversity of T4-like myoviruses was low in May, August, and November, with clear undersampling in May (except for May_S1_20). The degree of undersampling appeared less in August, and in November the total genetic diversity appeared to have been sufficiently sampled (Figure 6A).

For MCP viral assemblages, we observed a gradual increase in genetic richness from January to August (Figure 6B). Nearly all samples were sequenced to saturation, with the exception of three January samples (Jan_B8_1000, Jan_B16_500, Jan_B16_1000). The maximum genetic richness was observed in MCP assemblages sampled during August, after which genetic richness decreased slightly in the November samples. We observed a significant effect of depth on genetic richness for MCP assemblages, with genetic richness decreasing with increasing sample depth (ANOVA, Richness ~ Depth, F = 8.646 on 1 and 37 DF, *p* = 0.005622).

There was a significant difference in g23 diversity (H’) (Figure S3A,B) (ANOVA, *p* < 0.01) and community composition between sampling months and depth (ANOVA, *p* < 0.01). MCP diversity (H’) between sampling stations and month (ANOVA, *p* < 0.01) was also significant (Figure S3C,D), and MCP community composition differed significantly with depth (ANOVA, *p* < 0.01) and month (ANOVA, *p* < 0.01). Shannon diversity was highest during the polar night in both viral groups, with the lowest Shannon indices in May, thereafter increasing by August (Figure S3C).

**Figure 7:**
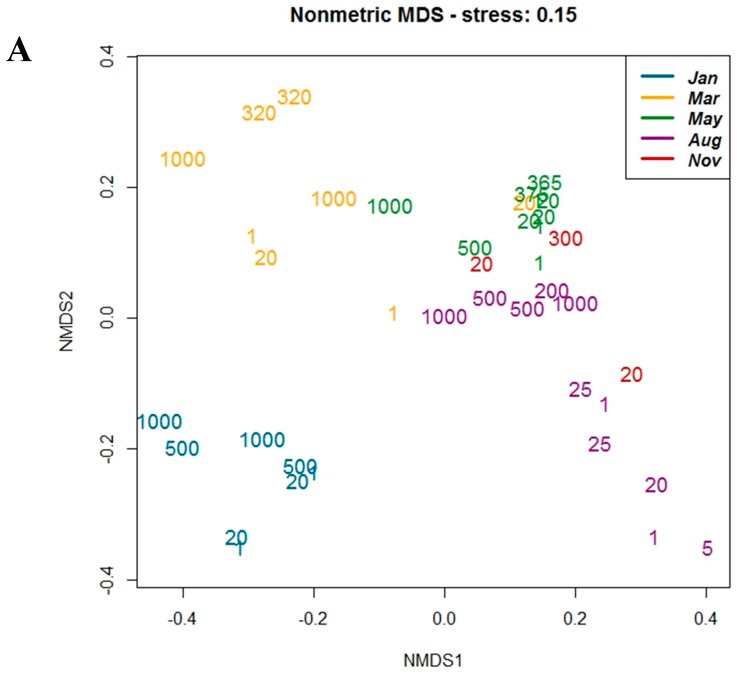

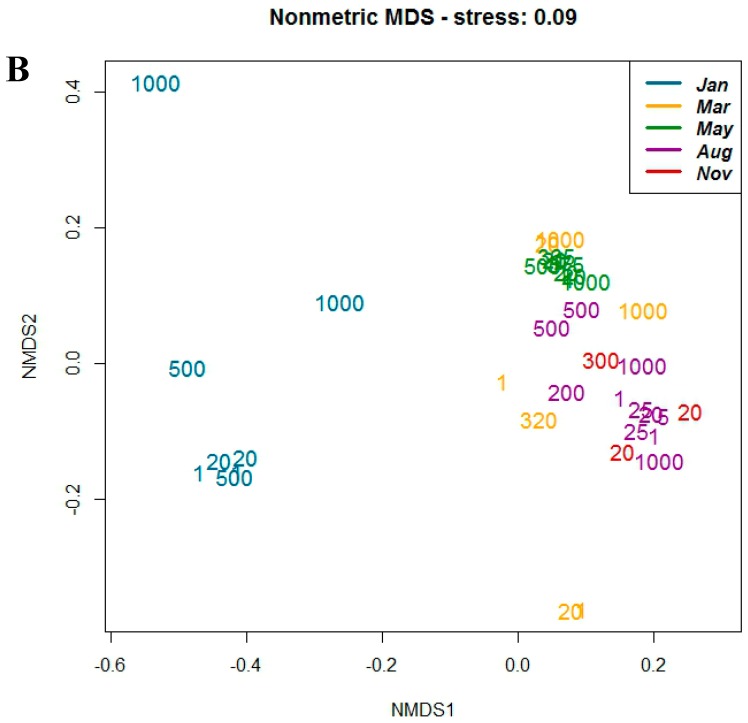
Nonmetric multidimensional scaling (NMDS) analysis of OTU diversity for (**A**) *g23* and (**B**) *mcp* genes. Numbers indicate sampling depth (m), and colors indicate sampling month. Samples clustered together based on similarity in OTU composition (Bray–Curtis dissimilarity), and the axes indicate separation between samples.

**Figure 8:**
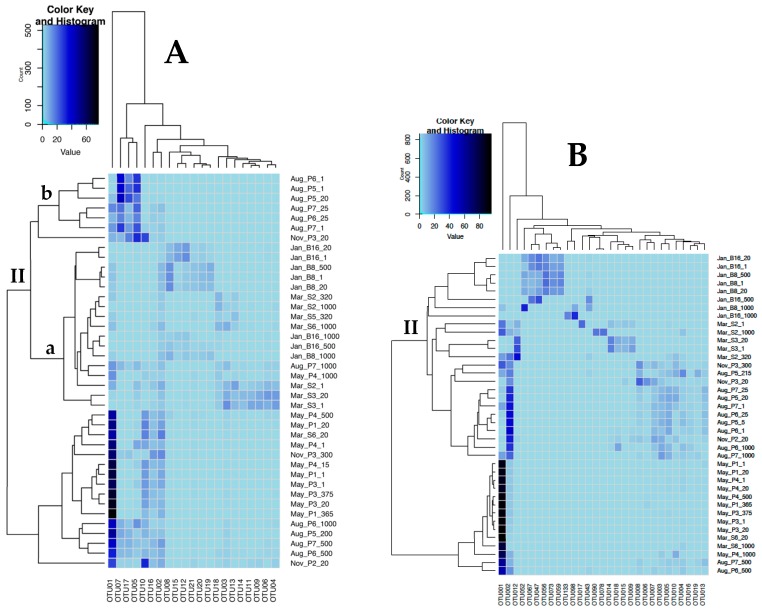
Two-way hierarchical clustering heat maps showing clustering of samples according to similarity in OTU composition. The analyses were based on the relative abundance of dominant OTUs (overall relative abundance >0.1%) in the individual samples. (**A**) *g23* and (**B**) *mcp* genes. The blue shading represents a continuous scale of OTU relative abundance from high (dark blue) to low (light blue).

Ordination analysis of OTU diversity (nonmetric multidimensional scaling (NMDS), Figure 7) demonstrated clear separation of sampling months for g23 (Figure 7A) and MCP (Figure 7B), with less contribution from sample depth. Comparison of the dominant g23 and MCP OTU assemblages by hierarchical clustering resulted in similar sample grouping, as shown in the tree topology at the top of the heat maps (Figure 8). For both genes in general, samples from the same time points grouped together, forming two main groups (I and II) consisting of the May and August samples (group I) and the August, November, January, and March samples (group II). Three exceptions were found for g23: May_P4_1000 clustered within group II, while March_St6_20 and November_St2_20 clustered within group I (Figure 8A). In general, g23 OTUs from August and November (St3_20) (IIb) grouped separately from samples from January and March (IIa). One exception was August_P7_1000, which showed the highest similarity to January and March samples (IIa).

The dominant g23 OTU (g23-OTU01) in all May samples also dominated August samples from 200 m in depth and deeper. Shallower August samples (<25 m) were mostly dominated by g23-OTU05, g23-OTU07, g23-OTU017, as well as g23-OTU01. The dominant g23 OTUs in the November samples were also OTUs that were found in high abundances in May and August. Dominant g23 OTUs in January and March were for the most part not found in high abundances at other sampling times.

**Figure 9:**
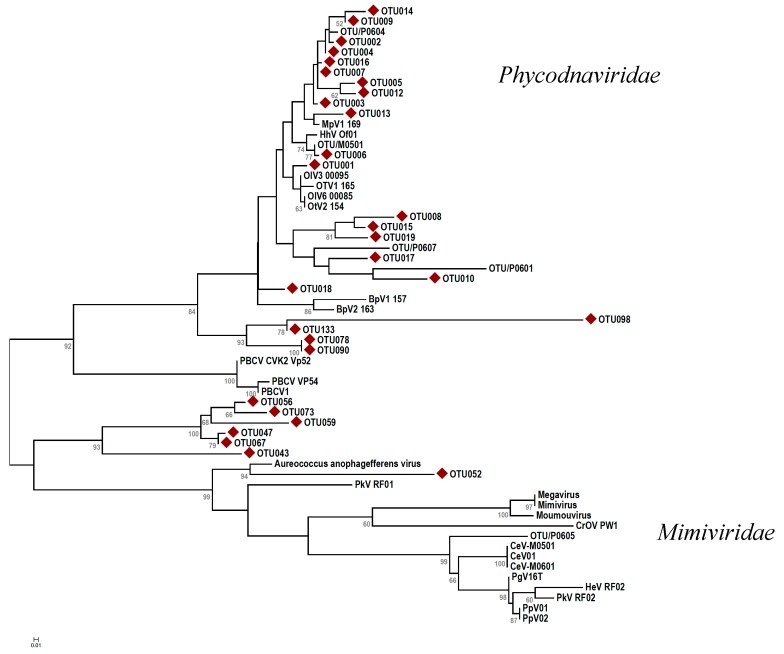
Maximum likelihood tree constructed from the 29 most abundant MCP OTUs (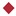). The evolutionary history was inferred using the maximum likelihood method based on the JTT matrix-based model [54] with 100 bootstraps. Branch lengths indicate the number of amino acid substitutions per site. Abbreviations: CroV = Cafeteria roenbergensis virus; Moumou = Moumouvirus goulette; Mimi = Mimivirus; Mega = Megavirus chiliensis; AaV = Aureococcus anophagefferens virus; PoV = Pyramimonas orientalis virus; PkV = Prymnesium kappa virus; HeV = Haptolina ericina virus; HhV = Haptolina hirta virus; CeV = Chrysochromulina ericina virus; PgV = Phaeocystis globosa virus; PpV = Phaeocystis pouchetii virus; PBCV = Paramecium bursaria chlorella virus; MpV = Micromonas pusilla virus; OsV = Ostreococcus sp. virus; OlV = Ostreococcus lucimarinus virus; BpV = Bathycoccus prasinos virus. Scale bar represents 0.2 substitutions per site.

Seventy-six percent of the MCP OTUs clustered within the Phycodnaviridae family, 21% made a separate branch between the Phycodna and Mimiviridae families, and only 3% fell within Mimiviridae (Figure 9). The two latter groups consisted only of OTUs from samples collected in January. MCP-OTU001 showed the highest similarity to cultured prasinoviruses infecting the Mamiellophyceae *Osterococcus lucimarimus* and *Osterococcus tauri*, and was the most abundant OTU detected in samples from all time points, with the lowest relative abundance in January thereafter increasing with a peak in May (Figure 8B). MCP-OTU002, the second most abundant OTU, showed the highest similarity to another Miellophyceae, Micromonas viruses (MicVs), and had the highest abundance in samples collected in August, followed by November, but was not detected in samples from January (Figure 8B). Nine OTUs (MCP-OTU003, -OTU004, -OTU005, -OTU007, -OTU008, -OTU010, -OTU013, -OTU016, -OTU019) had similar dynamics, with the highest relative abundance during the fall, and were not detected in samples from January (Figure 8B). Five of these (MCP-OTU003, -OTU007, -OTU005, -OTU004, -OTU016, and -OTU013) grouped together with both MCP-OTUT001 and MCP-OTU002, showing the highest similarity to cultured prasinoviruses (Figure 9). The last three OTUs (MCP-OTU008, -OTU010, -OTU019) made a separate cluster within the Phycodnaviridae family, distantly related to two environmental OTUs. MCP-OTU006, also clustering within the prasinovirus clade (Figure 9), was the only OTU with the highest relative abundance in the November samples, not detected in the samples from January and March and only at a very low relative abundance in some samples from May and August (Figure 8B). The polar night (January and March) was defined by eight OTUs (MCP-OTU009, -OTU012, -OTU014, -OTU015, -OTU017, -OTU018, -OTU078, -OTU090), with the highest relative abundance in samples from March, and nine OTUs (MCP-OTU043, -OTU047, -OTU052, -OTU056, -OTU059, -OTU067, -OTU073,-OTU098, and -OTU133), with the highest relative abundances in samples from January (Figure 8B). Four OTUs (MCP-OTU009, -OTU012, -OTU014, -OTU018) clustered within the prasinovirus clade, while MCP-OTU78 and -OTU90 made a deep branch within the Phycodnaviridae family, together with MCP-OTU113 and -OTU098 (Figure 9). Seven (MCP-OTU043, -OTU047, -OTU052, -OTU046, -OTU059, -OTU067, -OTU073) were unique to the January samples (Figure 7B). Six made a separate branch between the two viral families, but with highest similarity to Phycodnaviridae, while the last made a deep branch with the highest similarity to the *Aurecoccus anophagefferens* virus (Figure 9).

### 3.3. Correlation with Environmental Parameters

Canonical correspondence analysis demonstrated significant correlations between the g23 and MCP viral assemblages and sampling months, and to a lesser extent depth, with diversifying effects of temperature and virus abundance on g23 diversity (Figure S4A), or *Synechococcus* abundance on MCP diversity (Figure S4B).

## 4. Discussion

This study provides the first demonstration of seasonality and depth-driven diversification of virus assemblages in the Arctic marine environment. Furthermore, our results indicated that the dynamics of Arctic marine viral communities covaried with putative host populations [4,39], highlighting a role for the microbial loop in different productivity regimes in the Arctic [55]. Seasonal transitions in high-latitude regions are rapid (illustrated in Reference [56]), fueling dramatic changes to ecosystem structure and productivity across relatively short time spans. In the present study, the spring succession [6,37] and subsequent transition to low productivity at the onset of the polar winter were evidenced by shifts in the abundance and diversity of eukaryotic phytoplankton, heterotrophic prokaryotes, and viruses. Our metabarcoding strategy targeting two key virus groups thus revealed the dynamics of viruses potentially infecting hosts associated with primary (phytoplankton) and secondary (heterotrophic prokaryotes) production across an entire calendar year. Our sampling strategy, with discrete samplings at five time points (Figure 1), could not fully characterize the temporal viral dynamics. Nevertheless, our findings indicated that, from an ecological standpoint, sampling the month functioned well as a suitable proxy for time. To our knowledge, this represents the first integrated investigation of the seasonality of Arctic marine viral diversity, generating new knowledge about how microbial-driven ecosystem processes as a function of seasonality and depth in the Arctic effect the structure and activity of viral communities.

### 4.1. Linking Virus and Host Communities

Large dsDNA algal viruses [57] commonly co-occur with phytoplankton hosts and are estimated to be responsible for considerable turnover of primary production [58,59]. Comparisons of diversity profiles of host group (SSU rRNA) [60] and virus group (MCP) dynamics indeed suggest a strong link between host and virus diversity despite our inability to conclusively identify infective relationships. The pico- and nanophytoplankton fractions of microbial eukaryotes in Arctic marine ecosystems [37] were the likely hosts for the viruses targeted by our MCP metabarcoding approach [26]. *Micromonas pusilla* and *Bathycoccus prasinos*, for example, dominated phytoplankton communities in the May and August samples [60], and it was in these samples that we observed prasinovirus-like MCP OTUs to be dominant. In general, the genetic (OTU) richness of MCP virus assemblages increased with the progression of the spring-summer bloom (Figure 6), suggesting a radiation in large dsDNA algal virus diversity as potential host organisms (eukaryotic phytoplankton) increased in abundance and, by extrapolation, activity. The ecological importance of these phytoplankton groups for primary production in the Arctic marine environment is well known [38,61,62], thus indicating the likelihood that Arctic prasinophytes may be strongly top-down controlled by Phycodnaviruses and that this control, in addition to nutrient availability [5], may play an important role in regulating ecosystem productivity.

The second target for our metabarcoding strategy was the g23 major capsid protein gene of T4-like myoviruses. In contrast to the results for MCP virus assemblages, g23 virus assemblages decreased in genetic richness (Figure 6A) as their putative host populations (heterotrophic prokaryotes and cyanobacteria) increased in abundance (Figure 2 and Figure 3). This was suggestive of clonal amplification among viruses that were able to successfully infect actively growing hosts [63]. One relevant host group potentially targeted by T4-like myoviruses was the cyanobacterium *Synechococcus* sp. [25,39]. *Synechococcus* has generally been overlooked in studies of Arctic picoplankton diversity due to low water temperatures assumed to be inhibitory for growth of this common Atlantic taxon (but see References [64,65]). A study of *Synechococcus* diversity and growth from samples taken on the same cruise series described in this study demonstrated, however, that Arctic-adapted phylotypes of *Synechococcus* are abundant in this highly-Atlantified region of the Arctic [39]. Indeed, *Synechococcus*-like cyanobacteria were detected in our samples using flow cytometry, albeit at low abundances in January, March, and May. Abundances peaked in August at 2.1 × 10^4^ cells mL^−1^ and were followed by a decline in abundance to 1.0 × 10^3^ cells mL^−1^ in November. The dynamics of T4-like myovirus diversity observed in our samples, with genetic clustering of May, August, and November samples, suggested the tantalizing, albeit speculative, possibility of a host–virus linkage between Arctic *Synechococcus* assemblages and the T4-like myovirus diversity revealed by metabarcoding. Furthermore, the pattern of seasonality observed for g23 assemblages was comparable to the seasonal pattern observed for T4-like myovirus communities in coastal waters of western Norway [25,66], in which the seasonality of viral assemblages was strongly connected to changes in *Synechococcus* abundance. As no culture-based studies mapping host–virus interactions have been conducted, we cannot exclude the possibility that the pattern in T4-like myovirus diversity could also be explained by host–virus interactions between heterotrophic prokaryotes and bacteriophages.

### 4.2. Seasonality of Virus Assemblages

The March-to-May transition period represents the rapid onset of light and productivity that occurs annually in the Arctic. Our combined results showed that this transition was characterized by sharp drops in VPR (Figure 5) and virus diversity (Figure 8) simultaneously with increased abundances of heterotrophic prokaryotes (Figure 3), small VLPs, and large VLPs (Figure 4). Certain groups of heterotrophic prokaryotes, for example Gammaproteobacteria, respond rapidly to increased dissolved organic carbon (DOC) released during phytoplankton blooms [67]. Prokaryote groups, for example Gammaproteobacteria, whose growth rates rapidly respond to increased DOC availability during phytoplankton blooms, are more likely to be top-down controlled by viruses in a host-specific manner [9,11]. New virus production at the onset of the spring bloom may thus arise from infections of dominant, fast-growing hosts (heterotrophic prokaryotes), yielding relatively homogenous virus assemblages during the fast-growth spring period. In a parallel study from this cruise series [5], it was demonstrated that the highest cell-specific bacterial production rates were identified in surface waters during the May cruise, while the highest median bacterial production rates were measured in August. In a second parallel study describing prokaryote diversity from the same cruise series [4], it was revealed that known fast-growing heterotrophic prokaryote families, including the Oceanospirillaceae, Alteromonadaceae, and Flavobacteriaceae, dominated surface waters in May, while August surface samples were dominated by Halomonadaceae and Rhodobacteraceae [4]. As the dynamics of T4-like myovirus genetic diversity (Figure 8) quite accurately traces the changes in abundance of these putative host groups, it is reasonable to suggest that they may be trophically linked through host–virus interactions [25].

For MCP, the diversification and increase in evenness of virus assemblages and putative host groups [68] between the May and August and November samplings was in accord with previous observations that viral control of host assemblages promotes host community diversification. The co-occurring diversification of viral assemblages may be explained by increased host–virus encounters in the high-density bloom environment, promoting rapid viral co-evolution in pace with host diversification [69]. The increased MCP diversity observed in August persisted through the November sampling, suggesting that MCP viral diversity generated during the productive period defined microbial assemblages, including viruses, entering into polar winter. It remains uncertain, however, whether seasonal variation in Arctic marine virus assemblages are recurrent, as previously shown for fjord myovirus and host assemblages [25]. Interestingly, the increase in OTU diversity between May and August observed for MCP was not apparent in the g23 virus assemblages. One possible explanation for this observation is the potentially large host range of myoviruses, masking any fine-scale shift in HP dominance as system productivity shifted from dominance by primary production in May to dominance by secondary production in August.

Virus assemblages in January samples were the most genetically distinct of the five samples obtained and were characterized by the highest densities of viruses and the highest VPR. This raises speculation about the source of virus diversification during the polar winter, and to what extent shifts in virus assemblages observed during the dark months (January, March, and November) were due to new virus production. There are few studies focusing specifically on biological processes that take place in the marine environment during the polar night [70,71]. The high levels of metabolic activity observed for higher trophic levels in Kongsfjorden during polar night, for example, suggest the availability of stored, recycled, or advected organic matter. Furthermore, there is good evidence supporting the widespread occurrence of mixotrophy among eukaryotic phytoplankton [72], thus proffering the tempting possibility that new virus production may arise from infection of low-density, but highly diverse, overwintering mixotrophic protists. Alternatively, the stable cold and low-light conditions of the Arctic winter may indirectly promote virus persistence and accumulation through reduced decay rates ([73] and references therein, [74]).

### 4.3. Depth- and Winter-Driven Diversification

Our observations of relatively high VPRs during the low productivity winter period and at depth may be contrary to the common assumption that lysogeny is favored under conditions that foster low host cell abundance and activity [30]. The VPR is an index that has been used to assess the dominant viral strategy in a particular environment, with low VPRs indicative of lysogeny and high VPRs suggestive of host activity sufficient to support new virus production [75]. Our results, showing high relative abundance of free virus particles to putative host cells, is suggestive that viral lysis may yet occur during the polar winter (January, March, and November) and in deeper water layers, where system productivity is low [4,5]. Conversely, the low VPR observed in surface waters during the summer months (May and August) may suggest either the inability of new virus production to supersede high rates of HP proliferation [76] or may implicate high rates of lysogeny. These notions have, however, recently been challenged by inconsistent correlations of VPRs with environmental data, yielding both positive [77] and negative [78], as well as both weak [79] and strong [80], correlations between host activity, host abundance, or VPR. An alternative explanation for the high VPR observed during winter and at depth, and conversely the low VPR observed during the spring and summer months, may be a reduction in rates of virus decay during the low-light winter period [19]. This possibility is supported by our observations of relatively low VPRs in May and August, when viral lysis of host cells is anticipated to be at or near maximum rates due to rapid host growth. The high levels of biological activity, in combination with increased UV exposure in the photic zone, may have caused elevated virus decay rates. Although neither rates of lysogeny nor virus decay were assessed in this study, increased knowledge about physiochemical processes regulating the size and stability of virus assemblages in this strongly seasonal biome would make a welcome contribution to our understanding of Arctic marine microbial food webs.

While depth was also found to be a significant factor for marine virus assemblage diversification, its relative contribution to the total observed virus diversity was less pronounced, possibly due to downward vertical transport of particulate organic matter (POM) [5,81], providing a link between spring bloom production in surface waters and the seeding of microbial diversity and activity at depth during the low-productivity winter period. Although planktonic virus particles cannot by themselves sink, viral particles may be transported to deeper water layers either attached to POM [82] or inside sinking, infected host cells [83]. Indeed, POM in the form of both particulate organic carbon (POC) and particulate organic nitrogen (PON) was observed to be highest in the May samples [5], offering a potential mode of downward vertical transport for adhering virus particles generated in surface waters. This could explain the dominance of the same g23 OTU in May surface water and August samples from 200 m in depth and deeper. Downward flux may vary depending on size, water column stratification [4], grazing rates, and current strength (Reference [81] and references therein). There is evidence, for example, that formation of POM during bloom crash [84] may provide substrates for virus particle attachment and thereby downward flux [85]. The dominance of the colony-forming haptophyte *Phaeocystis pouchetii* at the time of the May sampling (M. Lund Paulsen pers. observ., UiB, Norway) likely contributed to a substantial downward flux of POM as the bloom declined, potentially transporting adsorbed cells and virus particles to deeper water layers. As sinking rates were not assessed in the present study, the interconnection between viral diversity in different water layers could not be assessed based on the present dataset.

### 4.4. Grazing by Microzooplankton

Interestingly, our results also provided an indication that prokaryote and protist communities from May to November may have experienced increased grazing predation by heterotrophic nanoflagellates in addition to viral pressure, in particular in surface waters (Figure 2). Although the balance of grazing versus viral lysis on microbial eukaryote and prokaryote assemblages could not be assessed based on the present data, previous studies of Canadian Arctic microbial communities have suggested that viral lysis may have a greater impact on bacterial communities than grazing pressure by heterotrophic flagellates during the winter [86]. Furthermore, prey switching between phytoplankton and prokaryotes before, during, and after the spring bloom may introduce variable grazing pressure on these communities [87]. Microzooplankton grazers have been found to be abundant in metabarcoding studies of Fram Strait seawater diversity [88]. One group of heterotrophic protists, the Picozoa [89], commonly found in Arctic eukaryotic metabarcoding studies [90], was also identified as one of the most abundant genotypes through metabarcoding analysis of eukaryote SSU rRNA genes [60]. Picozoa-like OTUs were present in all samples and at all depths: However the reduced Picozoan OTU evenness observed in the May samples [60] coincided with the observed reduction in MCP, and to a lesser extent g23, evenness among viral assemblages in May. This finding raises the intriguing possibility that the intense seasonality of the spring bloom regulates the diversity of all players in the food web, including viruses and their prokaryote and protistan hosts, although further research would be necessary in order to reveal specific host–virus linkages.

## Supplementary Material

The following are available at http://www.mdpi.com/1999-4915/10/12/715/s1: Figure S1, Boxplot analysis showing distance to centroids (OTU diversity) for 454 sequencing results in comparison to Illumina MiSeq sequencing results; Figure S2, Scatterplot showing heterotrophic prokaryote abundance versus small viruses (V1); Figure S3, Change in viral diversity with sampling month and depth; Figure S4, Canonical correspondence analysis (CCA) of OTU diversity for g23 and MCP; Table S1, List over samples analyzed in this study.
