# Supplementary material for: Seasonality Drives Microbial Community Structure, Shaping both Eukaryotic and Prokaryotic Host–Viral Relationships in an Arctic Marine Ecosystem
Source: Viruses. 2018 Dec 14;10(12):715. doi: 10.3390/v10120715 (PMC6315344; doi:10.3390/v10120715)
Supplement: Supplementary file 1 [file viruses-10-00715-s001.pdf]

Supplementary table and figures

Table S1. List over samples analysed in this study.

Month/station/ Depth (m)	Total MCP	Total g23	MCP[a]	% MCP[b]	g23[a]	% g23[b]	Number of unique g23 OTUs (0.03)	Number of unique MCP OTUs (0.03)
Jan_B8_1	ND[a]	236023	20112		160735	68,10	386	20
Jan_B8_20		44854	16080		30360	67,69	355	20
Jan_B8_500		279949	17948		187363	66,93	388	19
Jan_B8_1000		226038	6798		145118	64,20	566	13
Jan_B16_1		253413	16573		190995	75,37	362	21
Jan_B16_20		257986	18133		191412	74,19	417	18
Jan_B16_500		195738	11595		106205	54,26	461	10
Jan_B16_1000		130251	5568		73404	56,36	512	7
Mar_S2_1	519940	223388	123891	23,83	77296	34,60	274	26
Mar_S2_320	310861	111659	66379	21,35	36511	32,70	178	23
Mar_S2_1000	322079	388112	94949	29,48	118755	30,60	317	10
Mar_S3_1	356430	3245930	106663	29,93	935833	28,83	305	18
Mars_ S3_20	401552	2351712	108287	26,97	812321	34,54	296	25
Mar_S5_320	ND[b]	250940	ND[b]	ND[b]	45431	18,10	147	ND[b]
Mar_S6_20	317074	130038	99615	31,42	36841	28,33	133	10
M ar_S6_1000	682838	248060	96688	14,16	37696	15,20	289	8
May_P1_1	316021	97097	119333	37,76	16429	16,92	96	11
May_P1_20	263792	1592532	104098	39,46	375011	23,55	139	17
May _P1_365	342628	106208	145587	42,49	14886	14,02	80	9
May_P3_1	243233	88179	86737	35,66	19926	22,60	117	11
May_P3_15	245574	54524	89520	36,45	13099	24,02	90	10
May_P3_375	279547	93768	103778	37,12	25290	26,97	132	12
M ay_P4_1	352791	157705	128656	36,47	43103	27,33	155	15
May_P4_20	227105	97535	77564	34,15	20339	20,85	102	14
May_P4_500	408856	143480	152669	37,34	31451	21,92	213	11
May_P4_1000	478604	203913	61348	12,82	51310	25,16	327	7
Aug_P5_1	284254	101711	85361	30,03	39739	39,07	69	25
Aug_P5_ 20	363022	85519	91737	25,27	25175	29,44	80	26
Aug _P5_215	604089	198172	96711	16,01	22651	11,43	123	20
Aug _P6_1	426116	211045	126606	29,71	96192	45,58	114	35
Aug_P6_25	400091	128296	127359	31,83	35405	27,60	188	33
Aug _P6_500	984175	308057	237968	24,18	29811	9,68	244	24
Aug _P6_1000	508879	167645	125626	24,69	18404	10,98	105	24
Aug _P7_1	685901	412399	227625	33,19	118281	28,68	147	31
Aug _P7_25	442942	187368	158346	35,75	59537	31,78	192	29
Aug_P7_500	338532	180680	90946	26,86	49319	27,30	196	26
Aug _P7_1000	438283	179602	94707	21,61	31560	17,57	324	21
Nov_S2_20	237691	190704	75050	31,57	20825	10,92	182	36
Nov_S3_20	400508	155040	170323	42,53	14575	9,40	77	28
Nov_S3_300	219741	96905	51584	23,47	15874	16,38	103	32

ND[a] Not determined. Samples sequenced by 454 sequencing were not seperated according to barcodes before after trimming and quality check. Total number of reads before quality check were 232305 but included.

ND[b] No PCR products were generated by PCR amplification of this sample.

[a]Number of reads after trimming and chimera check
[b]Percentage of reads after trimming and chimera check

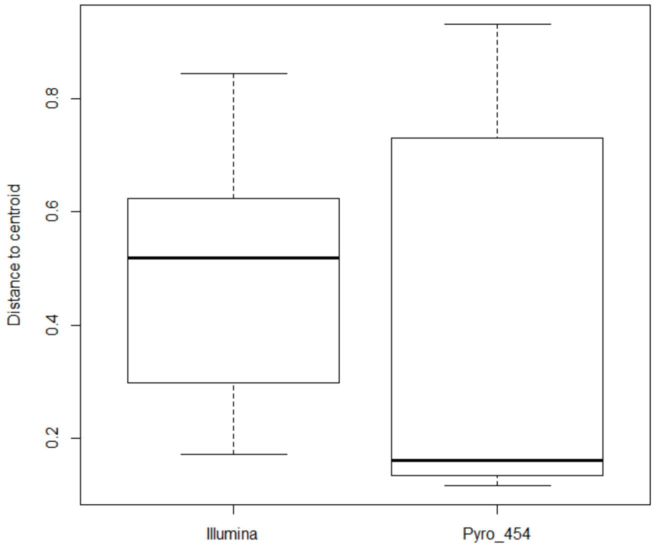

Figure S1. Boxplot analysis showing distance to centroids (OTU diversity) for 454 sequencing results in comparison with Illumina MiSeq sequencing results. No significant difference was observed between the two sequencing methods (ANOVA, p = 0.33862).

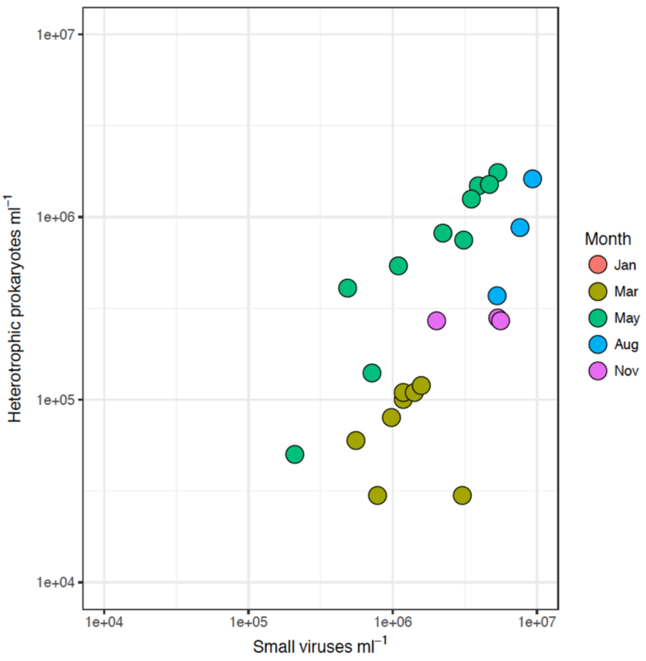

Figure S2. Scatterplot showing heterotrophic prokaryote abundance (y-axis) versus small viruses (V1) (x-axis). Note logarithmic axes. Sampling months are represented with different colours.

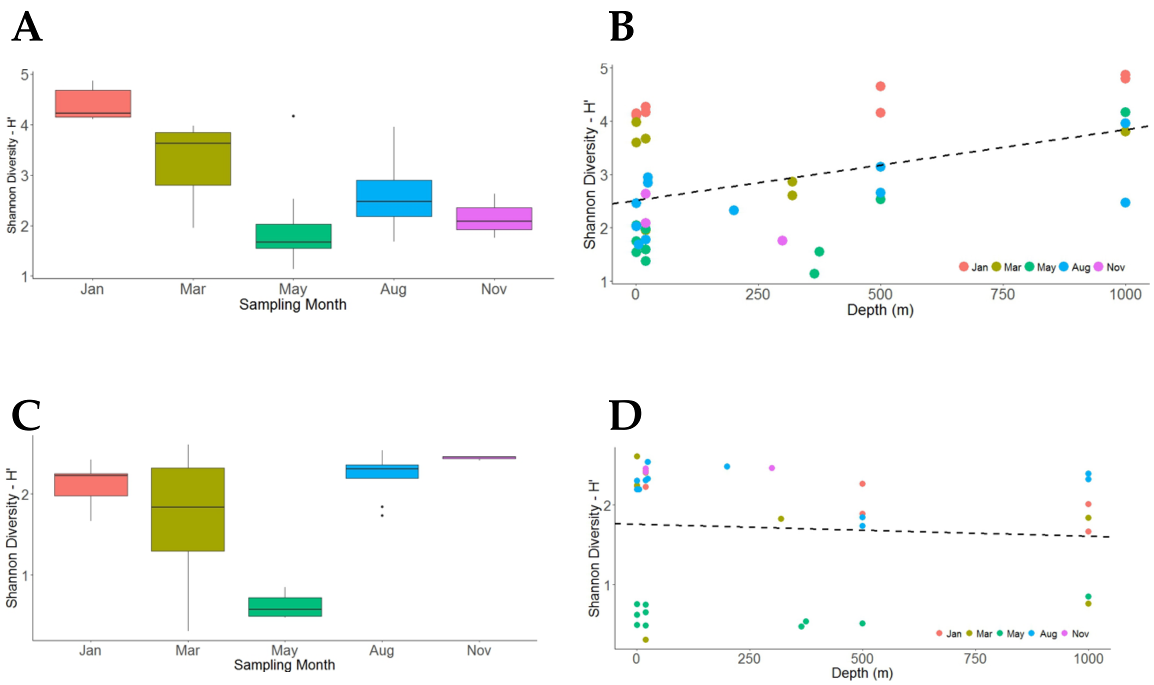

Figure S3. Change in viral diverstity (Shannon, H') with sampling month and depth. A) The boxplot show g23 OTU diversity present at different sampling months, with highest OTU diversity in January, and lowest in May. Black dot represent outlier. B) The graph illustrates changes in g23 diversity (H') with depth, and linear regression, indicated by dashed line, showed a significant change in diversity with increasing depth (ANOVA, $p < 0.01$). C) The boxplot show MCP OTU diversity at different sampling months, dots represent outliers. Lowest diversity was observed in May. D) The graph illustrates changes in MCP diversity (H') with depth. The linear regression (dashed line) did not show a significant change in diversity with increasing depth (ANOVA, $p = 0.66$).

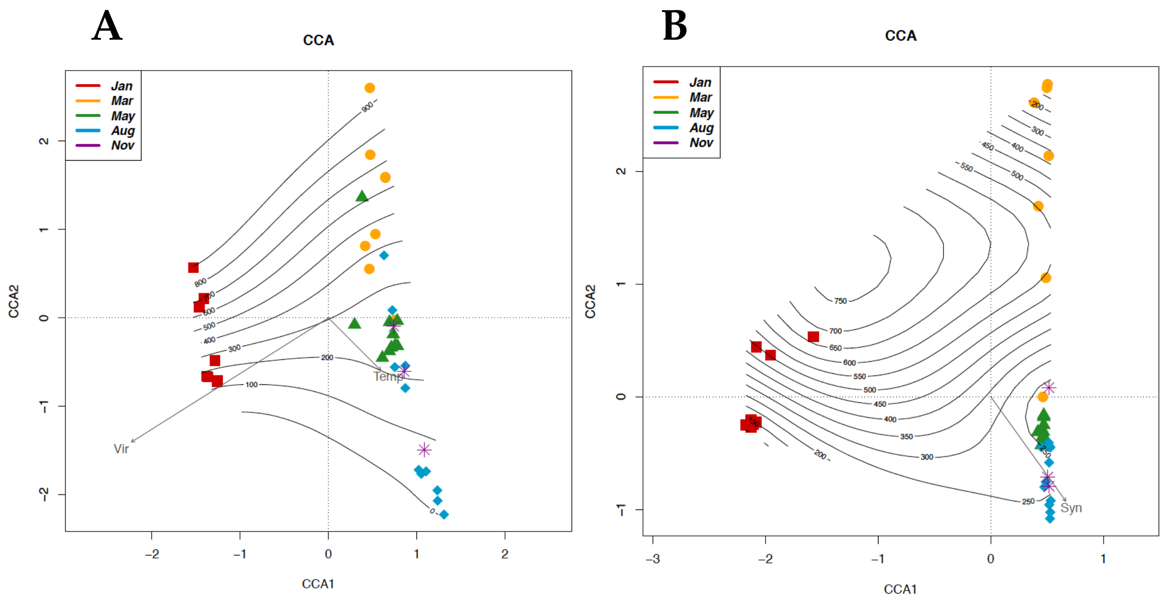

Figure S4. Canonical correspondence analysis (CCA) of OTU diversity for (A) g23 and (B) MCP showing the diversifying effects of depth (isobars), with significant environmental parameters indicated by vector arrows. Vir, virus abundance; Temp, water temperature; Syn, *Synechococcus* abundance.
